# Giant epiphrenic diverticulum: an unusual case from diagnosis to treatment

**DOI:** 10.1259/bjrcr.20210232

**Published:** 2022-02-21

**Authors:** Francesca Gorgoglione, Giulia Castorani, Nicola Palladino, Grazia Vittoria Orciulo, Gian Maria Ferretti, Diego Palladino, Anna Simeone, Marco Taurchini, Giuseppe Guglielmi

**Affiliations:** 1 Department of Clinical and Experimental Medicine, University of Foggia, Foggia, Italy; 2 Radiology Unit, IRCCS "Casa Sollievo della Sofferenza", San Giovanni Rotondo, Foggia, Italy; 3 Thoracic Surgery Unit, IRCCS "Casa Sollievo della Sofferenza", San Giovanni Rotondo, Foggia, Italy

## Abstract

Esophageal diverticulum is a rare disease caused by impairment of esophageal motility. The incidence is not known, due to lack of symptoms in many cases. Surgical treatment is reserved to symptomatic patients.

In this case report, we describe a rare case of epiphrenic esophageal diverticulum. A 61-year-old male with silent medical history, suffering severe chest pain had a CT scan showing a large esophageal diverticulum. The patient was referred to our hospital, IRCCS “Casa Sollievo della Sofferenza”, to complete pre-operative assessment with a CT scan and a Barium swallowing radiography, giving morphodimensional details of the diverticulum. Based on these findings, the surgeons have chosen the appropriate operative strategy. The surgeons adopted a laparoscopic access, completed with robotic-assisted laparotomy due to the morphology of the diverticulum.

Radiological evaluation is crucial in the diagnosis and in the treatment planning of symptomatic patients.

## Introduction

Esophageal diverticulum is a rare disease caused by impairment of esophageal motility. Esophageal diverticula are classified by the localization in: upper esophageal (Zenker: 70%), thoracic and mediastinal (10%) and epiphrenic diverticula (20%).^1^

The incidence is not known due to lack of symptoms in most cases. Diverticula should not be treated unless they are symptomatic. The most common symptoms are dysphagia, regurgitation, thoracic pain, and pulmonary manifestations related to aspiration. Barium swallow and upper endoscopy will help to establish the diagnosis while esophageal manometry allows to document esophageal motility disorders frequently associated to diverticula.^1^

We report the case of a patient diagnosed with a giant symptomatic epiphrenic diverticulum.

## Clinical presentation and radiological findings

**Figure 1.**
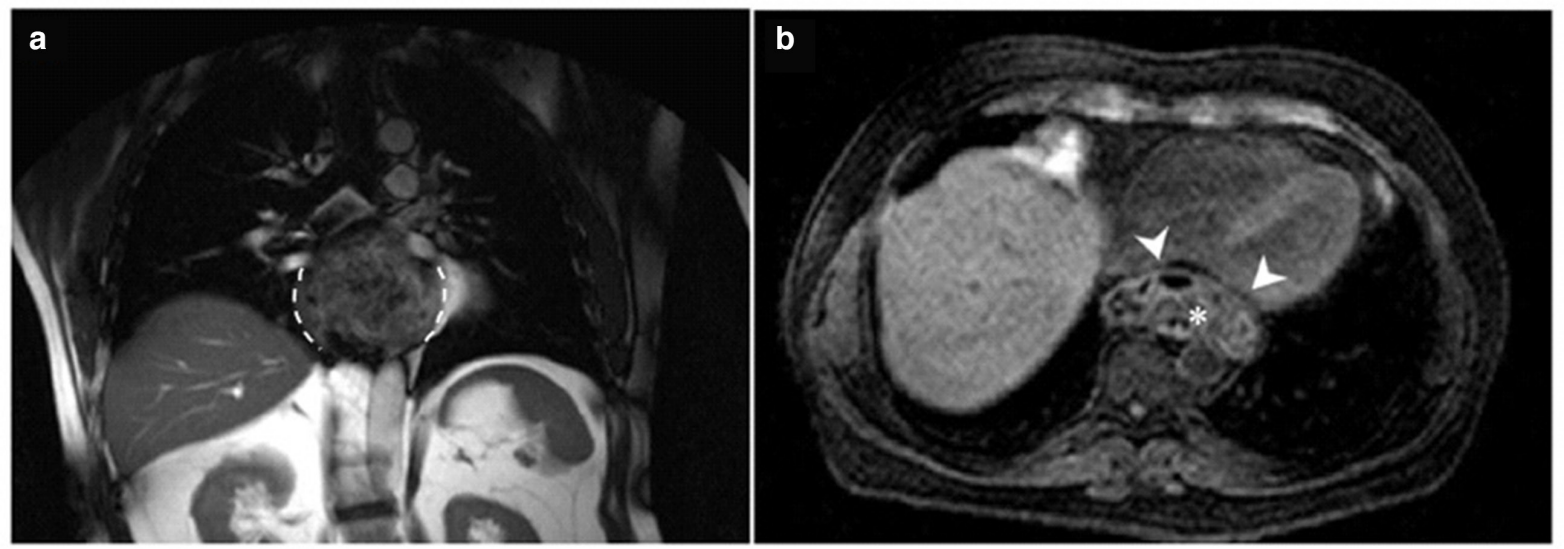
**(A**) Coronal *T*_1_W MRI demonstrated the left-sided large epiphrenic diverticulum of the esophagus (dashed lines); (B) axial thrive MRI showed the esophageal diverticulum compressing the left atrium and ventricle (arrowhead); it also evidenced the uneven content of the diverticulum, presumably food debris (asterisk).

**Figure 2.**
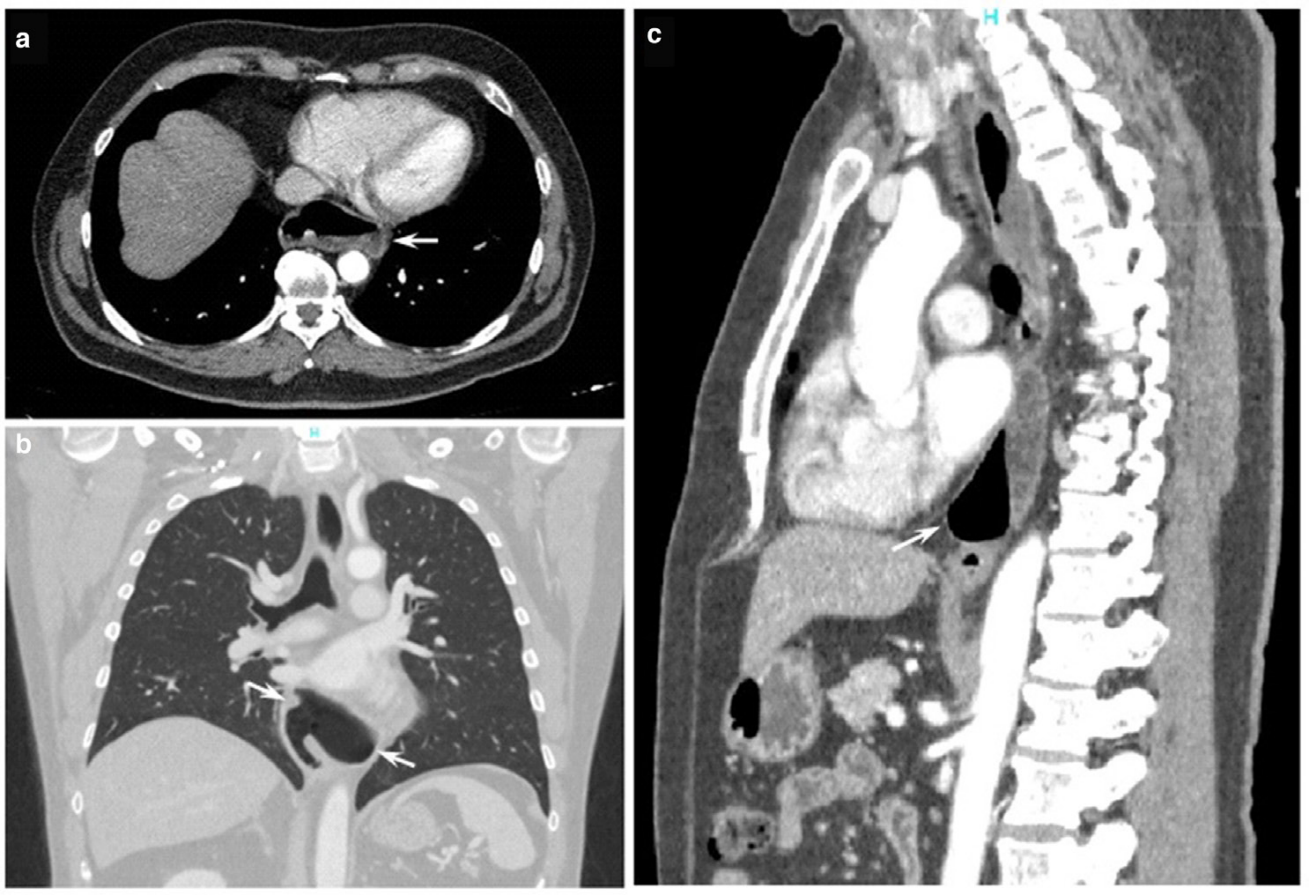
Pre-operative CT scan: June 2021. (A) Axial CE CT chest image in soft tissue window demonstrated a large outpouching on the left wall in the lower esophagus (arrow), with air-fluid level, due to the large esophageal diverticulum; (B) coronal CE CT chest image in lung window clearly showed the large left-sided esophageal diverticulum communicating with the esophagus (arrow); (C) sagittal CE CT chest in soft tissue window image showed the retrocardiac extension of the diverticulum (arrow).

**Figure 3.**
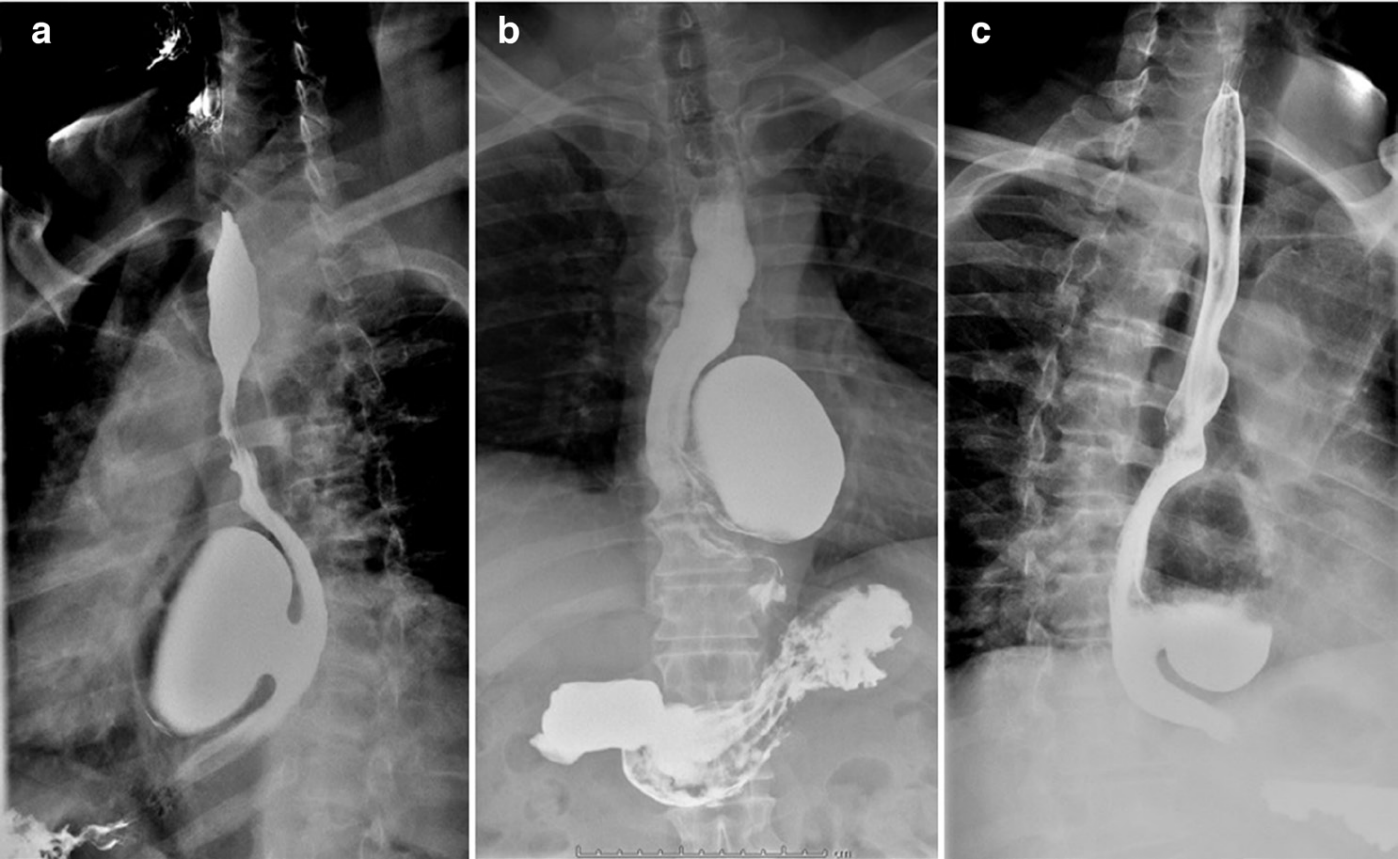
Barium swallowing X-ray showed the large epiphrenic esophageal diverticulum on the left anterior wall with 2,5 cm neck, in the right oblique projection (**A**), AP projection (**B**) and left oblique projection (C). AP, anteroposterior.

A 61-year-old male, without comorbidities, with severe retrosternal chest pain underwent chest basal CT with evidence of a giant epiphrenic diverticulum with a maximum diameter of 7,5 cm. Subsequently, he underwent MRI^2^ of the esophagus which confirmed the esophageal diverticular formation with moderate compressive effect on the atrium and left ventricle. (Figure 1) Given the closed relations with the heart, an echocardiogram was performed documenting impaired relaxation of left atrium and ventricle due to the compression of the diverticulum. In consideration of the symptoms, indications were given to surgical treatment of the diverticulum. The patient was then referred to our hospital, IRCCS "Casa Sollievo della Sofferenza", and admitted to the Thoracic Surgery Unit. During the admission, further radiological investigations were suggested to assess in details the dimensions, contents and other associated abnormalities. A CT scan was performed in the pre-operative phase showing a large epiphrenic esophageal diverticulum with a maximum diameter of 7,5 cm and a 2,5 cm wide neck, emerging about 7 cm far from cardias, partially overlapping the distal portion of the esophagus with air-fluid level, presumably referred to food debris (Figure 2). Barium swallow X-ray was performed and showed the transit through the upper gastrointestinal tract, giving details about the size, position and the dynamic of the diverticulum (Figure 3).

**Figure 4.**
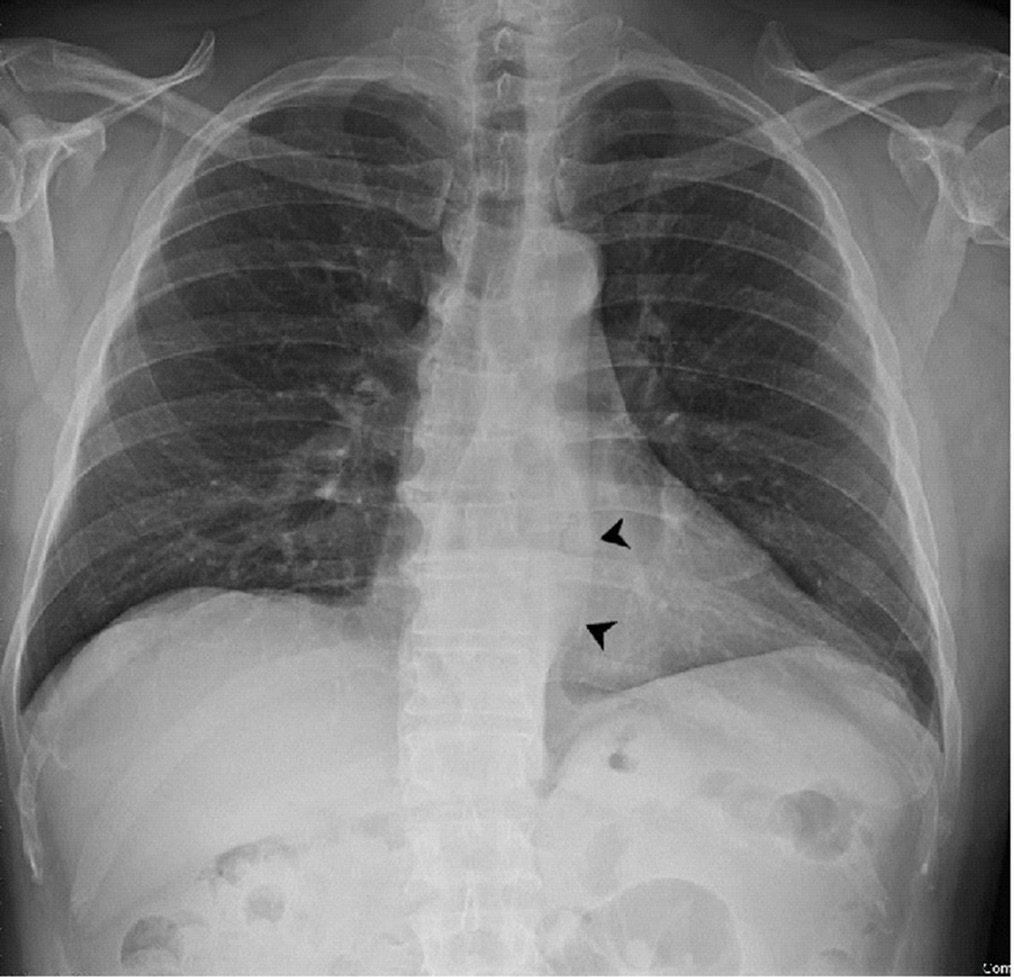
Pre-operative chest X-ray showing the wall of the diverticulum (arrowheads).

A pre-operative chest X-ray was performed to rule out eventual lung consolidation, showing the wall of the diverticulum (Figure 4).

Esophagogastroduodenoscopy documented normal esophageal mucosa with evidence of the known epiphrenic diverticulum in the lower third of the esophagus with a collar of about 2,5 cm. Esophageal manometry was performed unsuccessfully due to the width of the collar: during the exam, the catheter easily entered the lumen of the diverticulum instead of descending the esophagus to the sphincter.

## Treatment

The patient underwent transhiatal robot-assisted epiphrenic diverticulum preparation using a four-arm DaVinci^®^ Xi Robotic System.

The robotic system guarantees a much-improved vision thanks to the magnification and the depth perception of its 3D high-definition camera. In addition, the robotic instruments with seven degrees of freedom allowed to perform complex surgical maneuvers safely. For this reason, it was chosen to use this technique for the trans-hiatal preparation of the diverticular collar proceeding from the most caudal portion (located in correspondence of the esophageal hiatus) to the most cranial portion.

The most cranial portion of the diverticulum, located 7 cm from the esophageal hiatus, did not allow completing the preparation of the diverticular collar using the transhiatal robotic technique. Therefore, a muscle sparing minithoracotomy was performed in the right seventh intercostal space. After the preparation of the diverticular collar via thoracotomy, under endoscopic vision, diverticulectomy was performed with a tri-stapler mechanical stapler. The surgery was subsequently continued with robot-assisted abdominal surgery by performing Heller’s myotomy and Dor’s antireflux plastic using a four-arm DaVinci^®^ Xi Robotic System^3,4^

## Discussion

This case of giant epiphrenic diverticulum, with atypical clinical presentation, showed the importance of radiological investigations in the management of esophageal diverticula, given the eventual lack of significative symptoms in the diagnostic process. Symptoms differ from patient to patient. Many patients are asymptomatic and few show symptoms of mild dysphagia and reflux disease. Other patients may suffer from severe dysphagia, regurgitation, obstruction, heartburn, chronic cough, recurrent aspiration and pneumonia, cardiac arrhythmias, weight loss and halitosis.^5^ In the literature, cases of bleeding, carcinoma and/or perforation have also been reported.^6^ Differential diagnosis can include condition such as acid reflux, hiatal hernia or tumors, that can be differentiated based on the findings of standard work-up which is based on Barium swallow X-ray with supplemental evaluation including upper endoscopy to exclude malignancy and esophageal manometry to delineate any underlying motility disorder. Choosing the appropriate methods in the differential diagnosis process is essential in order to obtain useful morphodimensional and functional of information. Barium swallow X-ray plays a major role in the therapeutic management, giving morphological details in the pre-operative, such the location, and functional information in the post-operative, showing eventual leaking of contrast as a primary complication^7^ or a recurrence in the follow-up phase. Moreover, on the basis of the findings of the first post-operative esophagogram, without complications, the patient could resume oral feeding. CT scan is routinely performed in the pre-operative phase to evaluate the relations of the diverticulum with the nearby structures, depending on the location of the diverticulum, essential in surgery planning.^7^ Plain chest radiographs are commonly performed in the post-operative phase in order to evaluate the eventual presence of pneumothorax or lung consolidation resulting from the surgery and the thoracic drainages.

Complications due to the diverticulum itself can depend on the size, resulting in food retention, regurgitation or aspiration, or even to compression of nearby structures. Other worse complications are related to inflammatory process, such as ulceration, perforation, focal adhesions, abscesses, fistula and bleeding, and eventually malignant degeneration.^8^ Although these complications are extremely rare, a complete evaluation is recommended to decide the appropriate management. Post-operative complications include suture line leak, dysphagia and acid reflux.^9^

Effective treatment of the diverticula is surgery and is indicated for symptomatic patients only. Surgery strategies include not only diverticulectomy, but also a myotomy and fundoplication, or hiatal hernia repair if necessary.^10^ Hence, the treatment of epiphrenic diverticula is based on symptoms, not on size of diverticulum, focusing on the underlying motility disorder that is commonly present. Indications for surgery are worsening of symptoms such as dysphagia, regurgitation, or food retention, moreover on complications like compressive effect, aspiration pneumonia, perforation e-malignant transformation.^9^ The surgical approach can be different depending on the localization of the diverticulum. The actual trend includes minimally invasive procedure with laparoscopic/robotic procedure, rather than open surgical approach through laparotomy or left thoracotomy or a combined thoracoabdominal approach. Minimally invasive access depends on the localization of the diverticulum from the lower esophageal sphincter, resulting in transhiatal or transthoracic. This kind of procedure also includes video-assisted thoracic surgery^10^ and robotic-assisted devices such as “da Vinci^®^ Surgical System Robot”, improving the visualization of the site of interest compared to traditional laparoscopic/thoracoscopic approach.^3^ Given the pathophysiology of the diverticulum, of underlying motility disorders, myotomy is routinely employed to reduce the rate of recurrence. In absence of myotomy, it is estimated a recurrences rate of 20%, with leak rates of 24%. To avoid a leak, myotomy is commonly performed between 90 and 180 degrees from the diverticulectomy site. Usually, a myotomy is followed by a fundoplication to limit the post-operative reflux, which is observed in 9,5% of patients who received a Dor’s fundoplication, *vs* 48% after a partial fundoplication.^4^

## Outcome and follow-up

**Figure 5.**
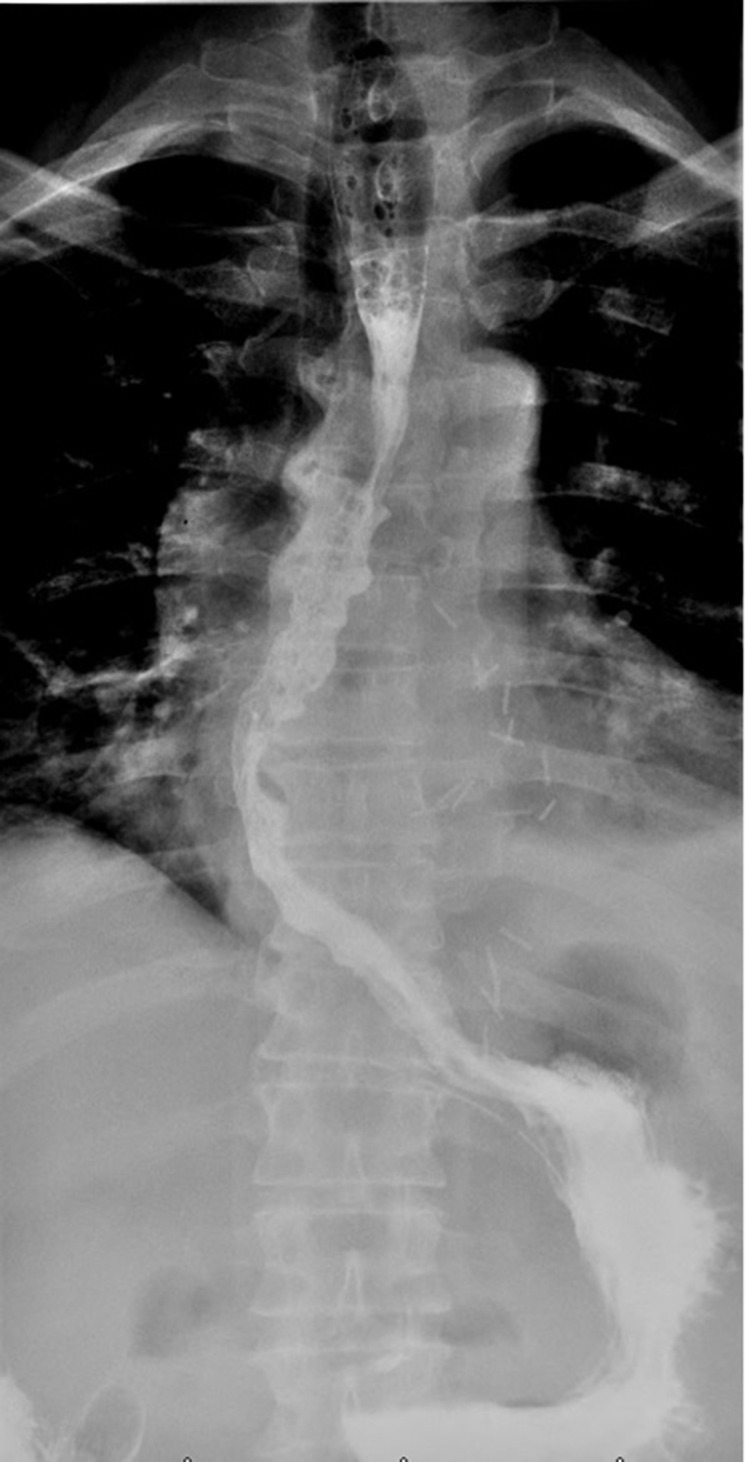
7 days post-surgery esophagogram with water soluble iodinated contrast medium showed no abnormal leaking of contrast.

Chest X-ray was performed post-operatively to evaluate lung re-expansion after thoracic surgical access. On the seventh post-operative day, the patient performed X-ray esophagogram with a water-soluble iodinated radiopaque contrast medium which documented regular transit of the contrast in absence of leak (Figure 5).

**Figure 6.**
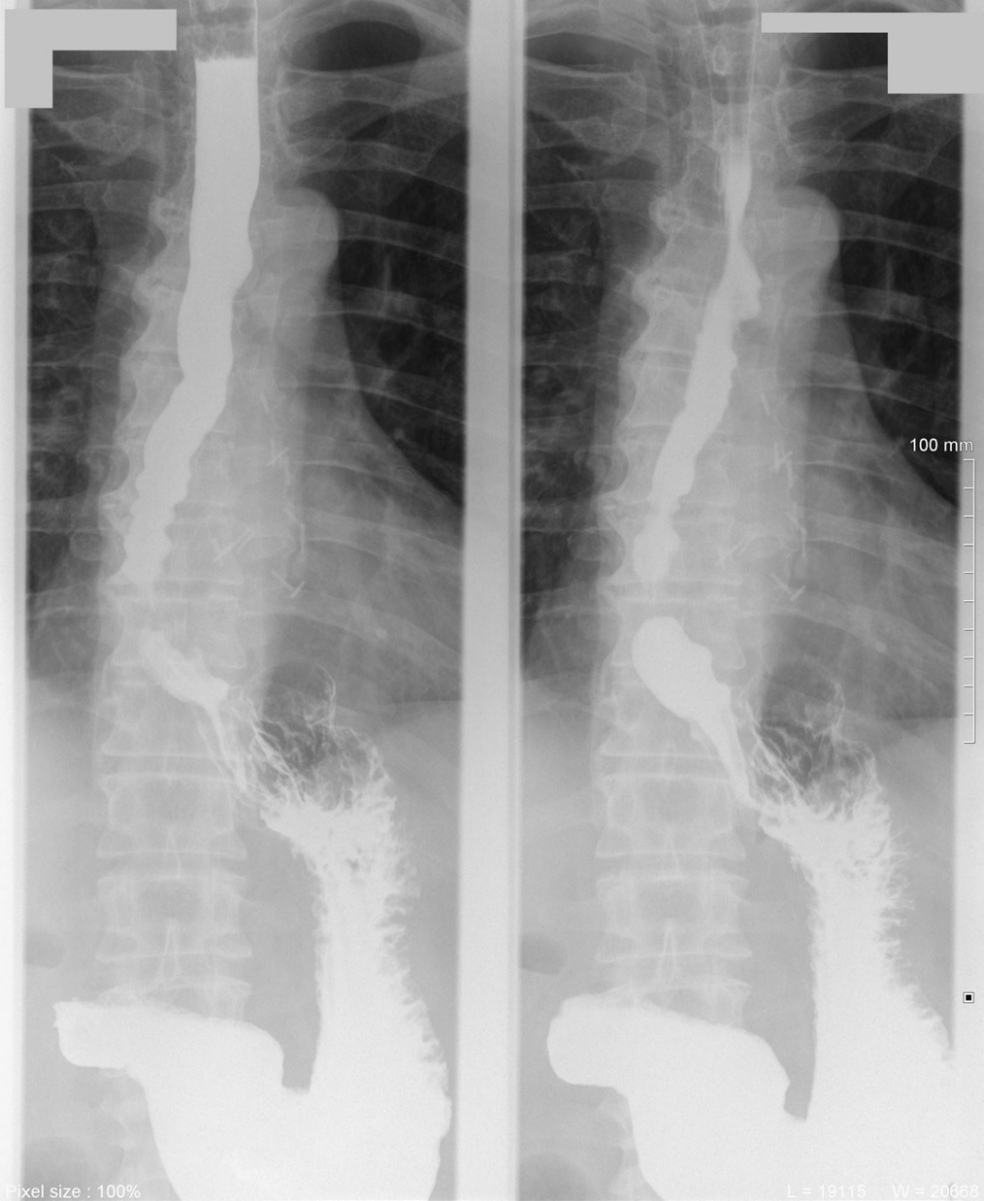
3 months post-surgery, Barium swallowing X-ray showed no recurrence of diverticulum.

In the following months, the patient was followed by a nutritionist who gradually changed his diet until he returned to a normal diet. The patient gained weight with an increase in lean mass. The patient reported a complete resolution of the symptoms without late complications from the surgery. 3 months after discharge, the patient underwent a further Barium swallow X-ray which documented normal transit of the contrast medium with no recurrence of epiphrenic diverticulum (Figure 6).

In conclusion, appropriate radiological investigations are crucial in the diagnostic and therapeutic management of patients with symptomatic esophageal diverticula.

## Learning points

- Epiphrenic esophageal diverticula are acquired pseudodiverticula, usually with underlying motility dysfunction.
- They are commonly asymptomatic and remain undiagnosed until symptoms such dysphagia, regurgitation, chest pain occur.
- Imaging techniques, such as Barium swallow X-ray and manometry, are gold-standard in classification e-functional evaluation, while CT scan gives morphodimensional information.
- Operative strategy based on radiological findings can optimize therapeutic management.
- Optimal treatment strategies and surgical approaches minimize the risk of complications both in the surgical and in the post-operative phase.

## References

[1] Clark SC, Norton SA, Jeyasingham K, Ridley PD. Oesophageal epiphrenic diverticulum: an unusual presentation and review. Ann R Coll Surg Engl 1995; 77: 342–45. 7486758 PMC2502434

[2] Leandri C, Soyer P, Oudjit A, Guillaumot MA, Chaussade S, et al. Contribution of magnetic resonance imaging to the management of esophageal diseases: a systematic review. Eur J Radiol 2019; 120: 108684. doi: 10.1016/j.ejrad.2019.108684 31563109

[3] Fisichella PM, Jalilvand A, Dobrowolsky A. Achalasia and epiphrenic diverticulum. World J Surg 2015; 39: 1614–19. doi: 10.1007/s00268-015-2950-7 25609118

[4] Hukkeri VS, Jindal S, Qaleem M, Tandon V, Govil D. Robotic transhiatal excision of epiphrenic diverticula. J Robot Surg 2016; 10: 365–68. doi: 10.1007/s11701-016-0595-7 27153837

[5] Bhandarwar AH, Tungenwar PN, Sawakare YM, Wagh AN, Patel CB, et al. Large epiphrenic diverticula: a rare case presentation. Clin Pract 2015; 5: 784. doi: 10.4081/cp.2015.784 26918097 PMC4745591

[6] Zaninotto G, Portale G, Costantini M, Merigliano S, Guirroli E, et al. Long-term outcome of operated and unoperated epiphrenic diverticula. J Gastrointest Surg 2008; 12: 1485–90. doi: 10.1007/s11605-008-0570-3 18622660

[7] Jang KM, Lee KS, Lee SJ, Kim EA, Kim TS, et al. The spectrum of benign esophageal lesions: imaging findings. Korean J Radiol 2002; 3: 199–210. doi: 10.3348/kjr.2002.3.3.199 12271166 PMC2713885

[8] Bennett B, Akhondi H. Epiphrenic Diverticula. StatPearls [Internet]. Treasure Island (FL): StatPearls Publishing; 2021. 32644536

[9] Herbella FAM, Dubecz A, Patti MG. Esophageal diverticula and cancer. Dis Esophagus 2012; 25: 153–58. doi: 10.1111/j.1442-2050.2011.01226.x 22335201

[10] Zanfrini E, Nachira D, Chiappetta M, Meacci E, Congedo MT, et al. Treatment of esophageal diverticula in uniportal video-assisted thoracoscopic surgery. Shanghai Chest 2019; 3. doi: 10.21037/shc.2019.01.03

